# Asymmetric cross-reactivity of nuclear receptors reveals an evolutionary buffer between estrogen and androgen signaling

**DOI:** 10.1016/j.isci.2026.117289

**Published:** 2026-08-19

**Authors:** Shintaro Yamazaki, Akhilesh B. Reddy

**Affiliations:** 1 Department of Systems Pharmacology & Translational Therapeutics, Perelman School of Medicine, University of Pennsylvania, Philadelphia, PA 19104, USA; 2 Institute for Translational Medicine and Therapeutics, Perelman School of Medicine, University of Pennsylvania, Philadelphia, PA 19104, USA; 3 Chronobiology and Sleep Institute (CSI), Perelman School of Medicine, University of Pennsylvania, Philadelphia, PA 19104, USA

**Keywords:** nuclear receptor, steroid receptor, ligand cross-reactivity, artificial intelligence, protein structure prediction, evolution, estrogen signaling, computational pharmacology

## Abstract

Nuclear receptor ligand interaction landscapes remain incompletely characterized, particularly at the scale of entire receptor families. We used Boltz-2, a structure-based deep learning framework for protein-ligand affinity prediction, to perform an unbiased survey of endogenous hormones across all human nuclear receptors. This analysis revealed an asymmetric pattern within steroid hormone receptors: estradiol showed broad predicted compatibility across multiple receptors, whereas other steroid hormones did not exhibit reciprocal compatibility with estrogen receptors. To interpret this pattern, we integrated structural modeling, evolutionary analyses, physiological contextualization, and prior experimental observations, which collectively led us to propose an evolutionary buffering framework for steroid receptor interactions. This study illustrates how large-scale affinity prediction can move beyond individual receptor-ligand pairs to reveal family-level interaction architectures, generate testable hypotheses, and guide future experimental investigation.

## Highlights

• Deep learning maps ligand interactions across the human nuclear receptor family
• Estradiol shows broad compatibility across steroid hormone receptors
• Structural and evolutionary analyses support ancestral estrogen compatibility
• All-by-all screening uncovers family-level interaction patterns

## Graphical abstract

**Figure:**
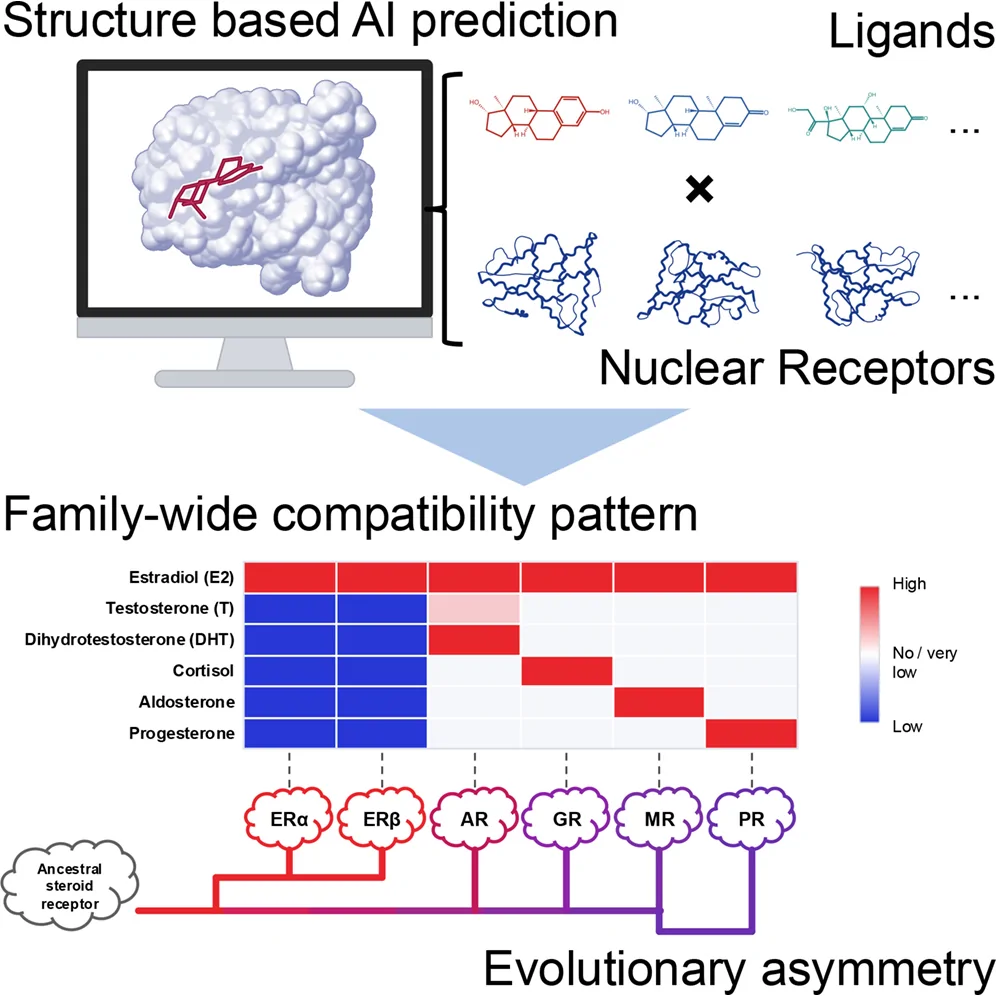

## Teaser

Molecular biology; Cell biology

## Introduction

Nuclear receptors (NRs) are ligand-regulated transcription factors that control diverse endocrine, metabolic, and developmental processes. Despite extensive study, NR interaction landscapes remain incompletely characterized, partly because many receptors are classified as orphan receptors lacking identified endogenous ligands.^1^ Of the 48 human NRs, approximately half have no firmly established physiological ligand.^1^^,^^2^ Even among receptors with known cognate hormones, potential secondary ligands and cross-reactive interactions are not comprehensively mapped, suggesting that functionally relevant crosstalk within the NR superfamily may remain underappreciated.^3^

Among NRs, the NR3 subfamily (steroid hormone receptors) provides a biologically and clinically relevant system to explore ligand cross-interactions. These receptors bind hormones derived from a common cholesterol-based biosynthetic cascade and share a conserved structural framework.^4^^,^^5^ The prevailing paradigm assumes one-to-one ligand-receptor specificity, with estrogen receptors (ERs: ERα and ERβ) responding to estrogens and androgen receptor (AR) responding to androgens. However, multiple empirical observations challenge this simple model. In prostate tissue, estradiol has been reported to suppress AR signaling and promote AR destabilization under physiological conditions,^6^ whereas in advanced prostate cancer, estradiol can activate AR signaling in permissive coactivator environments or ligand-binding domain mutations.^7^^,^^8^ These context-dependent effects indicate that receptor specificity is shaped by layered biochemical constraints and may not be fully captured by isolated ligand-receptor paradigms.

Evolutionary analyses further illuminate the origins of steroid hormone receptor specificity. Phylogenetic and functional analyses demonstrate that the common ancestor of modern steroid receptors (AncSR1) was estrogen responsive, exhibiting high affinity for aromatized ligands such as estradiol. Subsequent gene duplication and diversification produced the NR3 subfamily, including ERs, AR, progesterone receptor (PR), glucocorticoid receptor (GR), and mineralocorticoid receptor (MR).^4^^,^^9^ While these receptors evolved specificity for distinct pregnenolone-derived steroids, they retained a conserved ligand-binding scaffold inherited from an estrogen-sensitive ancestor.^5^ This evolutionary logic suggests that modern NR3 receptors may preserve latent compatibility with estrogens, despite their apparent ligand specificity.

Systematic evaluation of such cross-reactivity has been limited by experimental throughput. Classical approaches, including radioligand competition assays, surface plasmon resonance, and calorimetry, provide high-fidelity measurements but are necessarily targeted to pre-specified ligand-receptor pairs. Free-energy perturbation (FEP) methods offer physics-based computational alternatives but remain computationally intensive and impractical for exhaustive cross-family surveys. Recent advances in deep-learning-based protein-ligand modeling now enable broader interrogation. Boltz-2^10^ integrates structural co-folding with affinity prediction trained on large-scale experimental datasets, achieving performance comparable to FEP on held-out targets while operating at dramatically higher throughput. Importantly, as with all current scoring frameworks, Boltz-2 is most reliable for identifying relative ranking patterns within and across related proteins, whereas absolute cross-target comparisons remain subject to inter-protein scaling noise, a limitation common to docking and free-energy methods.^11^ These characteristics make Boltz-2 well suited for detecting directional trends and architectural asymmetries across receptor families at a scale not achievable experimentally with wet-lab assays alone.

Here, we performed an unbiased, all-by-all computational survey to map ligand-receptor interaction landscapes across the human NR superfamily. By integrating affinity prediction with structural modeling, receptor mutation analysis, ancestral receptor reconstruction, and simplified endocrine contextualization, we asked whether conserved evolutionary features of NR3 receptors contribute to asymmetric ligand cross-reactivity. Our results reveal a consistent pattern in which estradiol shows broad compatibility across the NR3 lineage, whereas non-aromatized steroids do not reciprocally engage ERs. This architecture aligns with established evolutionary models and provides a framework for interpreting context-dependent estradiol effects in physiological and pathological conditions.

## Results

### Comprehensive all-by-all affinity screen reveals asymmetric cross-reactivity in NR3 subfamily

To systematically map cross-reactivity across NRs, we performed an exhaustive all-by-all *in silico* affinity screen using full-length sequences of all 48 human NRs (NR0–NR6; Table S1) and a curated library of 54 endogenous ligands, including steroid hormones and intermediates, thyroid hormones, vitamin D metabolites, retinoids, fatty acids, and bile acids (Table S2). Ligand-receptor affinities and confidence scores were predicted using Boltz-2,^10^ generating a complete interaction matrix across the human NR repertoire (Data S1). For Boltz-2, predicted values are most informative for relative ranking trends within and across related receptors rather than absolute cross-target affinity magnitudes.

**Figure 1:**
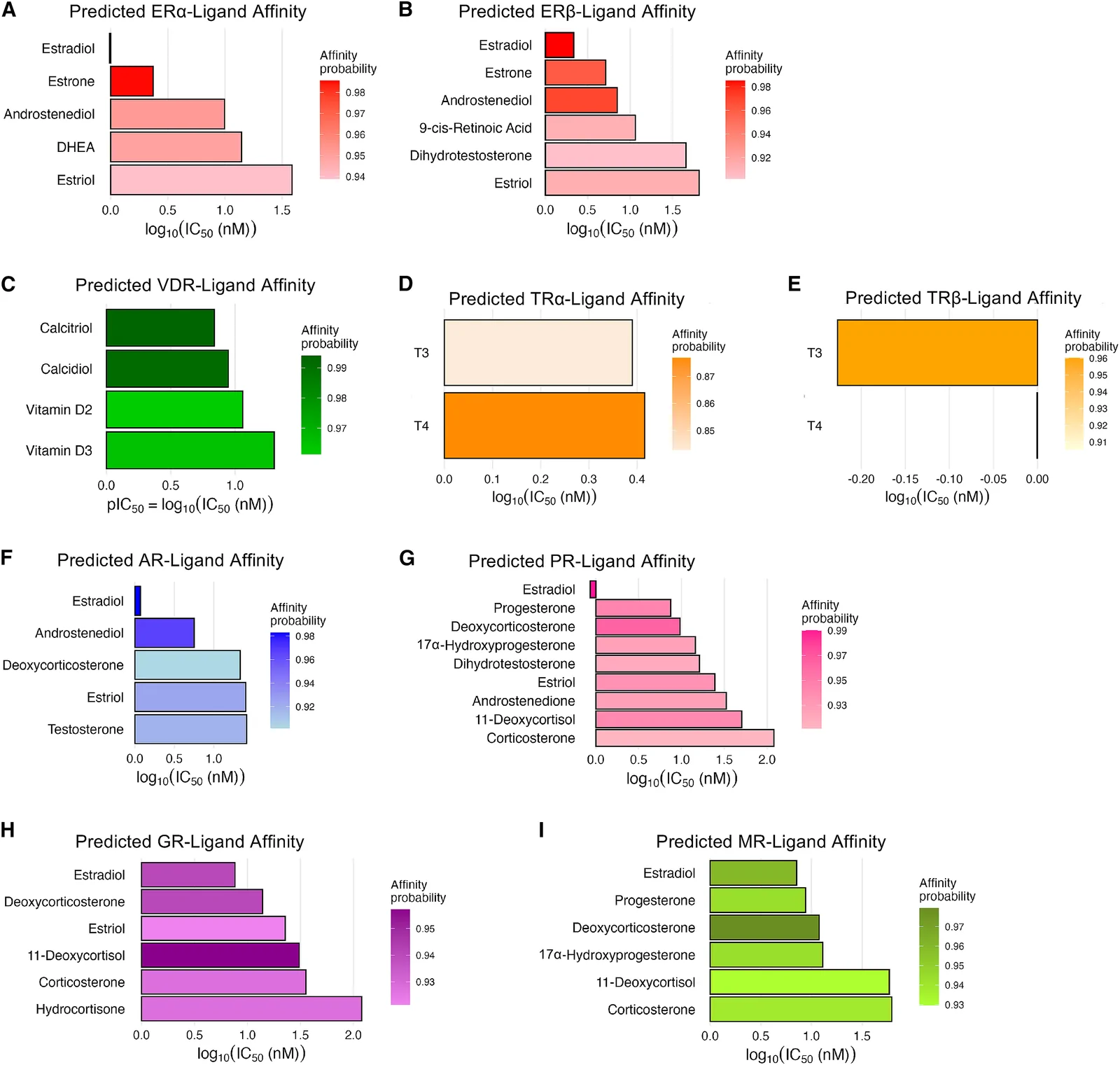
Boltz-2 predicts asymmetric 17β-estradiol compatibility across the NR3 subfamily (A) ERα binds 17β-estradiol (estradiol) with highest predicted affinity, followed by estrone and androstene-derived metabolites. (B) ERβ shows a similar rank order, with 17β-estradiol top-ranked, and modest binding predicted for 9-*cis*-retinoic acid and DHT. (C) VDR shows high affinity and confidence for calcitriol, followed by calcidiol and vitamin D2/D3, matching known biology. (D) TRα ranks T3 over T4, but neither ligand exceeds the confidence threshold (>0.90). (E) TRβ shows higher confidence binding for T3 over T4, consistent with TRβ’s known ligand preference. (F) AR shows strong predicted compatibility with 17β-estradiol with high model confidence, exceeding that of androgens. (G) PR shows top binding to 17β-estradiol and progesterone, with notable affinity for multiple steroid intermediates including deoxycorticosterone and 17α-hydroxyprogesterone. (H) GR shows predicted 17β-estradiol compatibility comparable to that of glucocorticoids such as cortisol, corticosterone, and their precursors. (I) MR shows high-confidence affinity for estradiol, alongside aldosterone and related mineralocorticoid precursors. Bar plots show predicted ligand affinities (*x* axis: log_10_ (IC_50_ [nM])) for each receptor. Lower values indicate stronger predicted affinity. Color intensity represents Boltz-2 prediction confidence (scale bars at right), with darker bars indicating higher model certainty (maximum = 1.00).Only ligands with prediction confidence >0.90 are displayed (except for TRα, which had no ligands exceeding this threshold). IC_50_ and confidence scores are derived from the Boltz-2 model, a deep learning framework trained on receptor-ligand structural data to jointly predict binding strength and interaction likelihood.

As an internal benchmark, the screen recovered known cognate ligand preferences with appropriate within-receptor rank ordering. ERα/ERβ showed strongest predicted affinity to 17β-estradiol (E2), followed by estrone (E1) and estriol (E3), consistent with known estrogenic potency^12^ (Figures 1A and 1B). The vitamin D receptor (VDR) preferentially bound calcitriol (1,25-dihydroxyvitamin D3) over calcidiol (25-hydroxyvitamin D3) and vitamin D2^13^^,^^14^ (Figure 1C). Similarly, thyroid hormone receptors (TRs: TRα and TRβ) preferentially bound T3 over the prohormone T4^15^^,^^16^ (Figures 1D and 1E). Neither VDR nor TRs showed high-confidence binding to steroid hormones, including estradiol or androgens. Additional comparisons with representative experimental binding data and isolated ligand-binding domain analyses further evaluated the computational framework (Figures S1A and S1B; Table S3; Data S2).

Beyond these expected interactions, the global screen revealed a directional pattern in NR3 ligand-ranking outputs. E2 was predicted to engage not only ERs but also several 3-ketosteroid receptors (AR, PR, GR, and MR), where it ranked among the higher scoring ligands beyond cognate ligands (Figures 1F–1I). The reciprocal pattern was not observed: canonical ligands for AR/PR/GR/MR (e.g., testosterone/dihydrotestosterone [DHT], progesterone, cortisol, and aldosterone) did not rank comparably within ERα or ERβ under the same conditions (Figures 1A and 1B). The interaction landscape therefore appears directional (E2 engages multiple steroid receptors, whereas non-aromatized steroids remain excluded from ERs). Given the accurate recovery of cognate ligand hierarchies described earlier, this estradiol cross-reactivity is unlikely to reflect general ligand promiscuity in the model but instead appears specific to the NR3 lineage (Data S1).

### E2 is predicted to be compatible with the AR, consistent with prior biochemical evidence

Among the predicted cross-reactive interactions, the E2-AR pair was particularly notable. Within the AR-focused ligand ranking, E2 ranked above testosterone under identical model conditions (Figure 1F), whereas testosterone showed negligible predicted affinity for ERs (Figures 1A and 1B). While absolute affinity values should not be interpreted as quantitatively precise across targets, this relative ordering indicates that E2 is predicted to be structurally compatible with the AR ligand-binding pocket.

Direct biochemical studies support the existence of E2-AR interactions, albeit at lower affinity than canonical androgens.^17^ In prostate cell contexts, Yeh et al. demonstrated that estradiol can drive AR transcriptional activity in the presence of the AR coactivator ARA70, implying productive occupancy of the AR ligand-binding pocket under permissive cofactor conditions.^7^ Competitive binding experiments have reported E2-AR binding IC_50_ values in the low-nanomolar range in relevant assay settings.^7^ Conversely, androgen binding to ERα is extremely weak relative to estradiol,^12^^,^^18^ reinforcing the directional asymmetry observed in our screen.

Extending beyond AR, E2 also ranked among the top predicted ligands within PR, GR, and MR (Figures 1G–1I), supporting the interpretation that E2 displays an unusual degree of compatibility across 3-ketosteroid receptors, whereas those receptors’ principal ligands do not reciprocally engage ERs (Figures 1A, 1B, and 1F–1I).

### E2 is structurally compatible with a conserved steroid-recognition pocket of the AR

**Figure 2:**
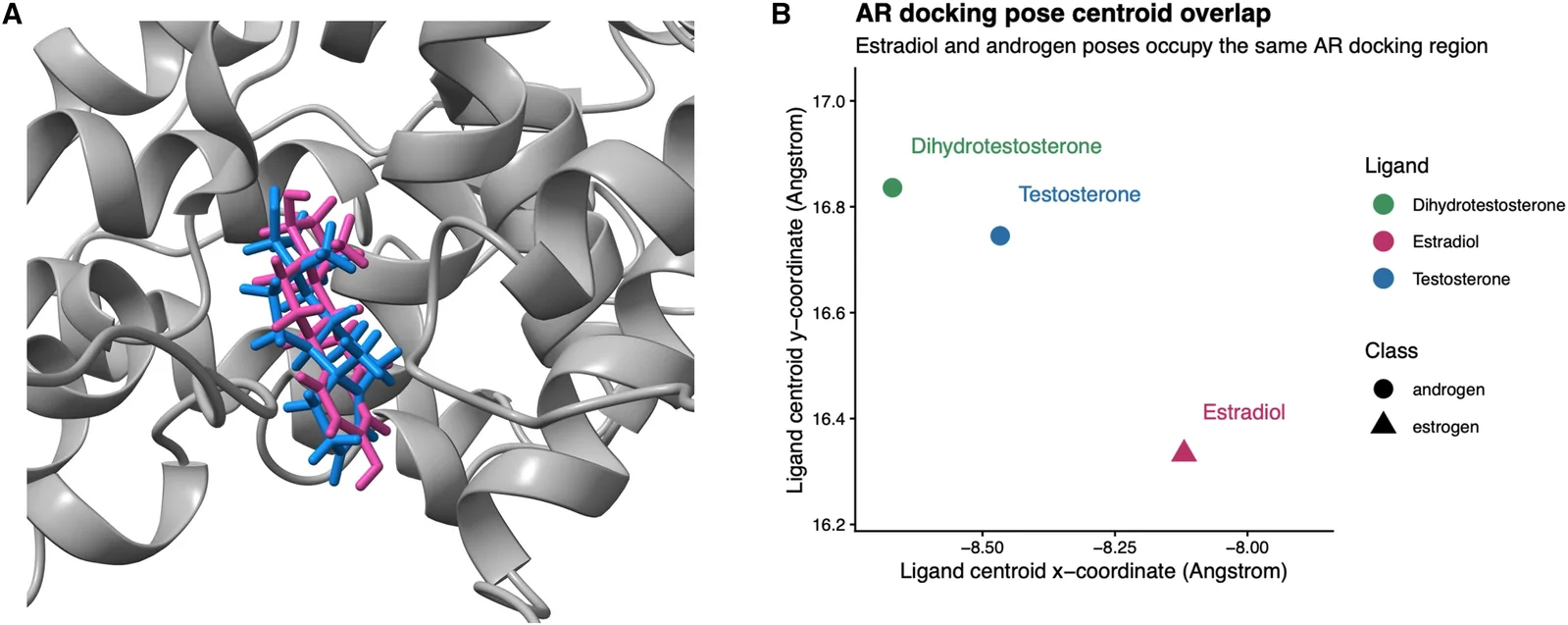
17β-estradiol is accommodated within the canonical AR pocket used by endogenous androgen ligands (A) Structural superposition of 17β-estradiol (magenta) and testosterone (blue) docked within the ligand-binding domain (LBD) of the androgen receptor (AR), shown as a gray ribbon representation. Both ligands adopt highly overlapping poses within the canonical AR binding cavity, indicating that estradiol is accommodated without a distinct binding mode. Docking was performed using AutoDock Vina with a blind search grid encompassing the AR LBD (40 × 40 × 40 Å) centered near the receptor center of mass, and the highest scoring pose located within the canonical pocket was selected for analysis. Pose visualization was performed in ChimeraX. The shared occupancy of this pocket suggests a conserved steroid-recognition architecture in AR underlying asymmetric ligand compatibility. (B) Docking centroid comparison including 17β-estradiol (estradiol), testosterone, and dihydrotestosterone. Docking scores were similar (17β-estradiol: −7.37 kcal/mol; testosterone: −7.52 kcal/mol; dihydrotestosterone: −7.48 kcal/mol), and pose centroids overlapped closely (17β-estradiol-testosterone: 0.624 Å; 17β-estradiol-dihydrotestosterone: 0.829 Å). Docking supports shared-pocket accommodation.

Importantly, docking did not require an alternative or mutation-exclusive binding mode. Using a validated docking protocol (Figure S1C), E2 and testosterone docked within the canonical AR ligand-binding pocket with highly overlapping poses (Figure 2A). Quantitative analyses demonstrated that E2 exhibited docking scores and pocket occupancy comparable to the canonical androgen ligands testosterone and DHT with similar AutoDock Vina scores (E2: −7.37 kcal/mol, testosterone: −7.52 kcal/mol, and DHT: −7.48 kcal/mol) and overlapping pose centroids (E2: 0.624 Å and E2-DHT: 0.829 Å; Figure 2B). These data support a conserved steroid-recognition architecture in AR that is permissive for both aromatized and non-aromatized steroids despite their chemical differences. Critically, the docking results argue against a specialized or mutation-dependent binding mode for E2; instead, E2 exploits an existing pocket whose geometry predates strict androgen specificity and was later refined for non-aromatized steroids. This structural interpretation is consistent with evolutionary reconstructions indicating that modern NR3 ligand-binding pockets derive from an estrogen-sensitive ancestral architecture that was subsequently tuned during specialization for non-aromatized steroids.

### AR T877A tunes pre-existing estradiol compatibility rather than creating a novel interaction

**Figure 3:**
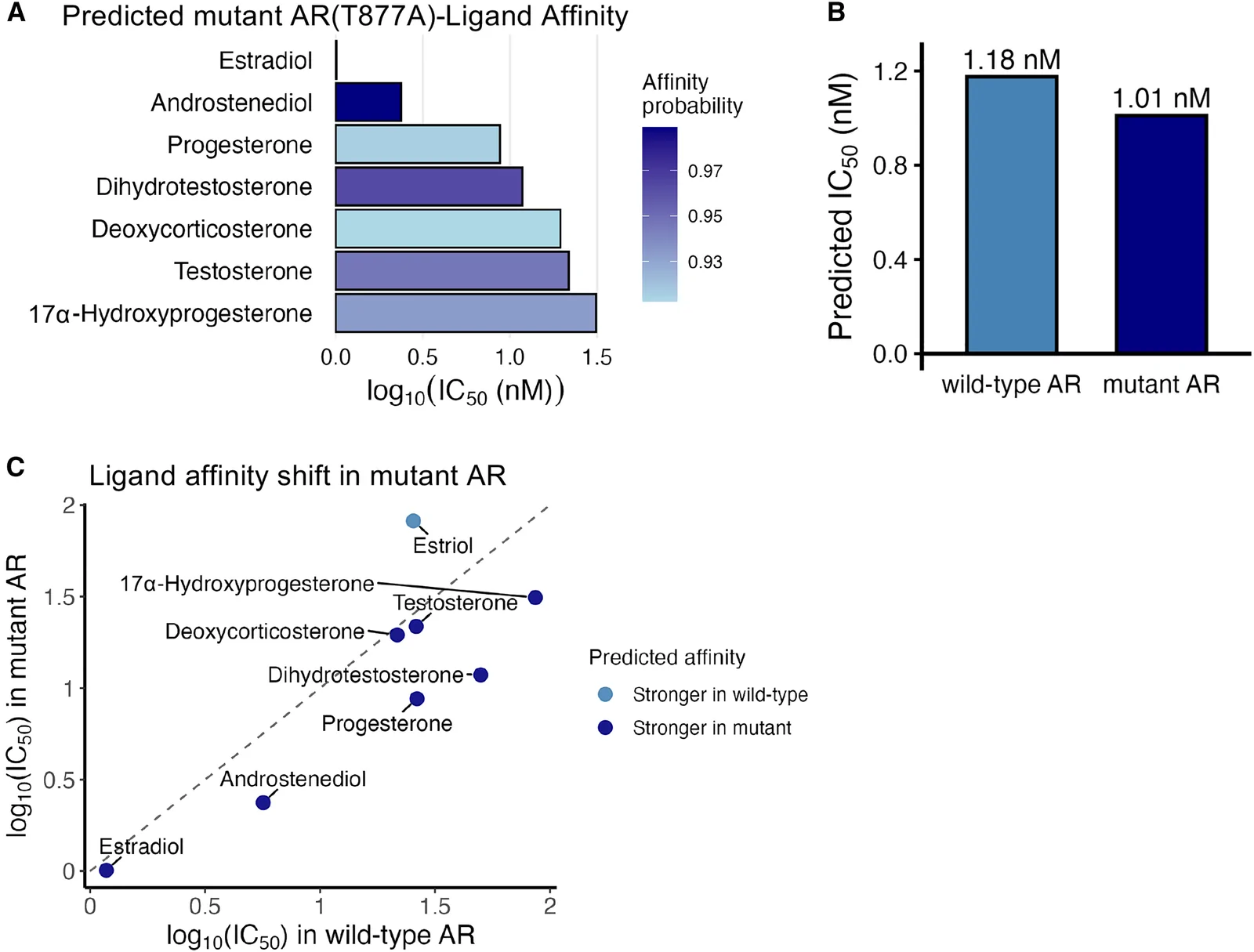
AR T877A modestly shifts a pre-existing ligand-compatibility landscape (A) Predicted mutant AR (T877A)-ligand affinity. Boltz-2 identifies multiple steroids with high predicted affinity and confidence (probability ≥0.93) toward T877A AR, including 17β-estradiol, androstenediol, progesterone, dihydrotestosterone, deoxycorticosterone, testosterone, and 17α-hydroxyprogesterone. Across the ligand panel, T877A produces modest and broadly distributed predicted shifts. (B) Boltz-2 estimates similar 17β-estradiol IC_50_ values for wild-type and T877A AR (about 1.18 and 1.01 nM). (C) Scatterplot comparing wild-type and mutant AR predicted IC_50_ values (log_10_). Points below the diagonal indicate stronger predicted affinity in the mutant.

To determine whether disease-associated AR mutations create new E2 responsiveness or instead repurpose an existing interaction, we analyzed the T877A AR mutant, a clinically relevant variant associated with ligand promiscuity in prostate cancer.^17^^,^^19^ Across the ligand panel, T877A produced modest, broadly distributed affinity gains, rather than selectively favoring a new ligand class (Figures 1F and 3A).

Notably, E2 remained among the higher ranking ligands for AR in the mutant context (Figure 3A), with predicted IC_50_ values remaining similar between wild-type and mutant AR (Figure 3B). This suggests that T877A modestly tunes a pre-existing structural compatibility rather than inventing a new binding mode.

The pattern of affinity shifts across ligands supports this interpretation. T877A does not uniquely elevate affinity for a specific ligand class. Instead, it subtly stabilizes ligand engagement across the steroid spectrum (Figure 3C). Together with the shared-pocket docking results, this suggests that the mutation primarily tunes receptor conformational stability and coactivator permissiveness, rather than fundamentally altering ligand recognition or binding specificity.

### Ancestral receptor analysis indicates estradiol as the evolutionarily oldest NR3 ligand

**Figure 4:**
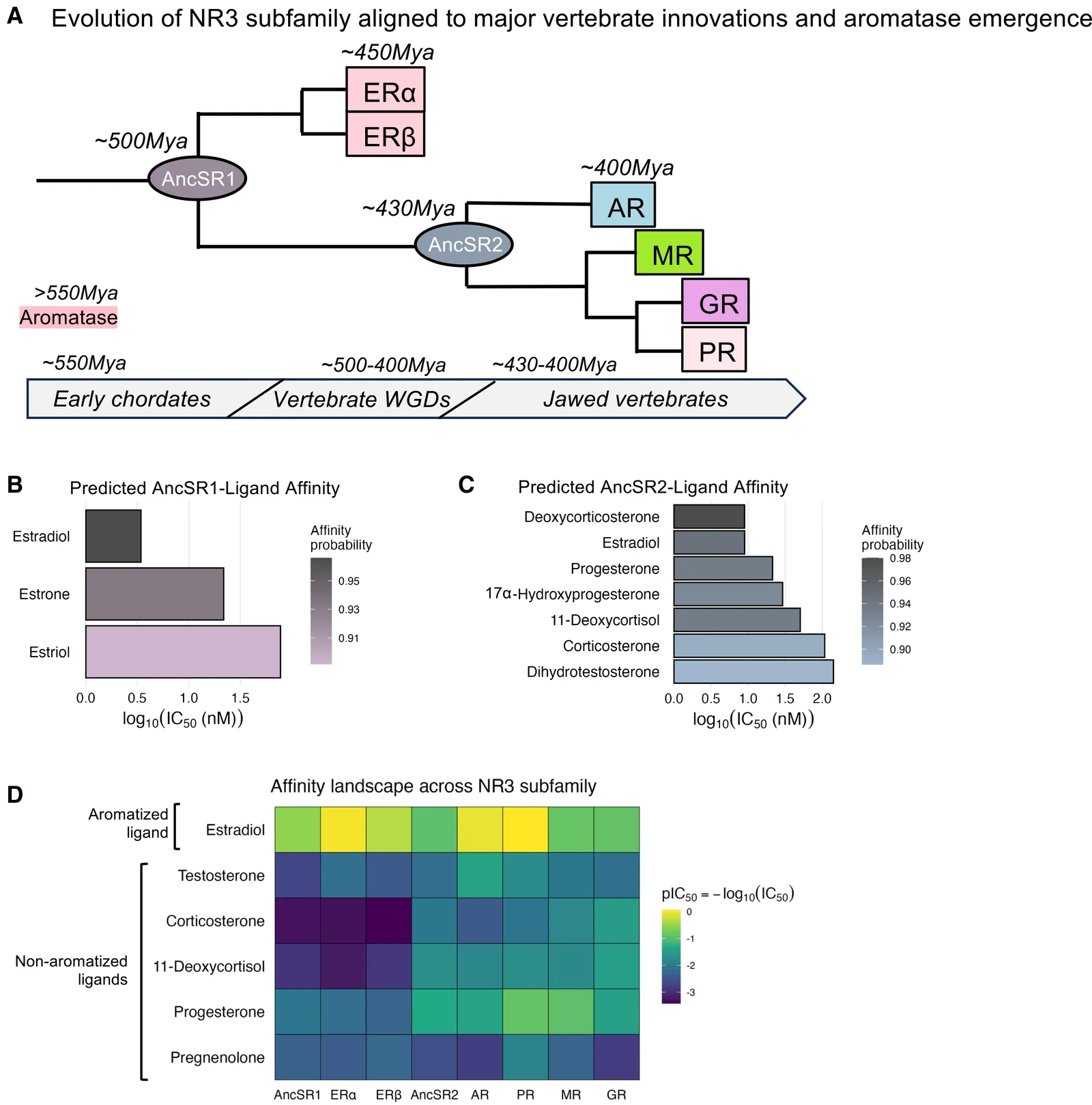
Ancestral receptor analysis is consistent with progressive diversification of steroid recognition across the NR3 lineage (A) Phylogenetic relationships among NR3 receptors with reconstructed ancestral nodes indicated. AncSR1 (∼500 million years ago) represents the experimentally supported estrogen-sensitive ancestral steroid receptor. A subsequent duplication during early vertebrate whole-genome duplication events (WGDs) produced ERα and ERβ (∼450 million years ago). A later divergence generated AncSR2 (∼430–400 million years ago), the common ancestor of AR, PR, GR, and MR. This framework is based on previously published evolutionary reconstructions. (B and C) Predicted ligand affinities for AncSR1 and AncSR2 (probability ≥0.874), respectively. AncSR1 shows strong predicted affinity for aromatized estrogens, consistent with experimentally supported ancestral estrogen sensitivity. AncSR2 displays a broader predicted affinity landscape that includes both aromatized and non-aromatized steroids. (D) Predicted affinity landscape across ancestral and modern NR3 receptors for key nodes of the steroidogenic cascade. Tile color represents predicted binding affinity expressed as pIC_50_ = −log_10_(IC_50_), with lighter (yellow) colors indicating stronger predicted affinity and darker colors indicating weaker predicted affinity. Ligands are ordered according to their biosynthetic hierarchy (pregnenolone → progesterone → corticosteroids/androgens → estradiol). Across the predicted affinity landscape, estradiol remains a recurrent high-scoring ligand across ancestral and modern receptors, whereas other steroids show progressively diversified receptor preferences following NR3 lineage expansion. These observations are consistent with evolutionary models in which modern receptors retain features of an estrogen-sensitive ancestral architecture while diversifying specificity for non-aromatized steroid ligands.

To place the observed asymmetry in evolutionary context, we examined predicted compatibilities for reconstructed ancestral receptors, AncSR1 and AncSR2^20^ (Figure 4A). The ancestral affinity profiles reveal a clear pattern: E2 maintains consistently high predicted compatibility across both ancestral (Figures 4B and 4C) and modern receptors (Figures 1A, 1B, and 1F–1I), whereas other steroids display progressively diversified compatibility profiles following NR3 lineage expansion (Figure 4D).

AncSR1, representing the earliest reconstructed steroid receptor, shows strong preference for estradiol and limited responsiveness to non-aromatized steroids (Figure 4B). AncSR2 begins to acquire compatibility with non-aromatized ligands, but E2 remains a high-ranking ligand (Figure 4C). In modern receptors, specificity for testosterone, progesterone, and corticosteroids increases, yet ancestral estradiol compatibility is retained (Figure 4D). Importantly, the emergence of aromatase (CYP19A1) predates the diversification of modern NR3 receptors^21^ (Figure 4A), establishing estradiol as an ancient, pre-existing ligand in early vertebrates. Subsequent NR evolution therefore occurred in the context of an already available estrogenic end product, rather than requiring *de novo* ligand invention.

This evolutionary trajectory provides a plausible context for the asymmetric compatibility pattern observed in extant receptors. Although estradiol is the terminal product of steroid biosynthesis, it is the evolutionarily oldest ligand in the NR3 lineage.^4^ As the NR3 lineage expanded, younger receptors progressively diversified to recognize upstream non-aromatized steroids in the biosynthetic cascade, while retaining ancestral sensitivity to the downstream end-product estradiol (Figures 4A and 4D). In contrast, ERs are optimized for aromatized estrogen recognition, never evolved responsiveness to non-aromatized steroids, thereby reinforcing an asymmetric specialization.

Together, these data indicate that modern AR and related NR3 receptors do not appear to acquire estradiol compatibility adventitiously but instead retain and repurpose an ancestral estrogen-compatible architecture while evolving specificity for newer steroid ligands.

### Ligand competition modulates the contribution of E2 to receptor occupancy

The compatibility screen and structural analyses suggest that E2 is structurally compatible with the AR ligand-binding pocket. However, receptor-ligand compatibility represents only one layer of endocrine biology. *In vivo*, receptors are simultaneously exposed to multiple competing ligands whose relative availability changes dynamically across tissues, physiological states, and endocrine phases as a result of hormone production, plasma binding proteins, and tissue metabolism.

**Figure 5:**
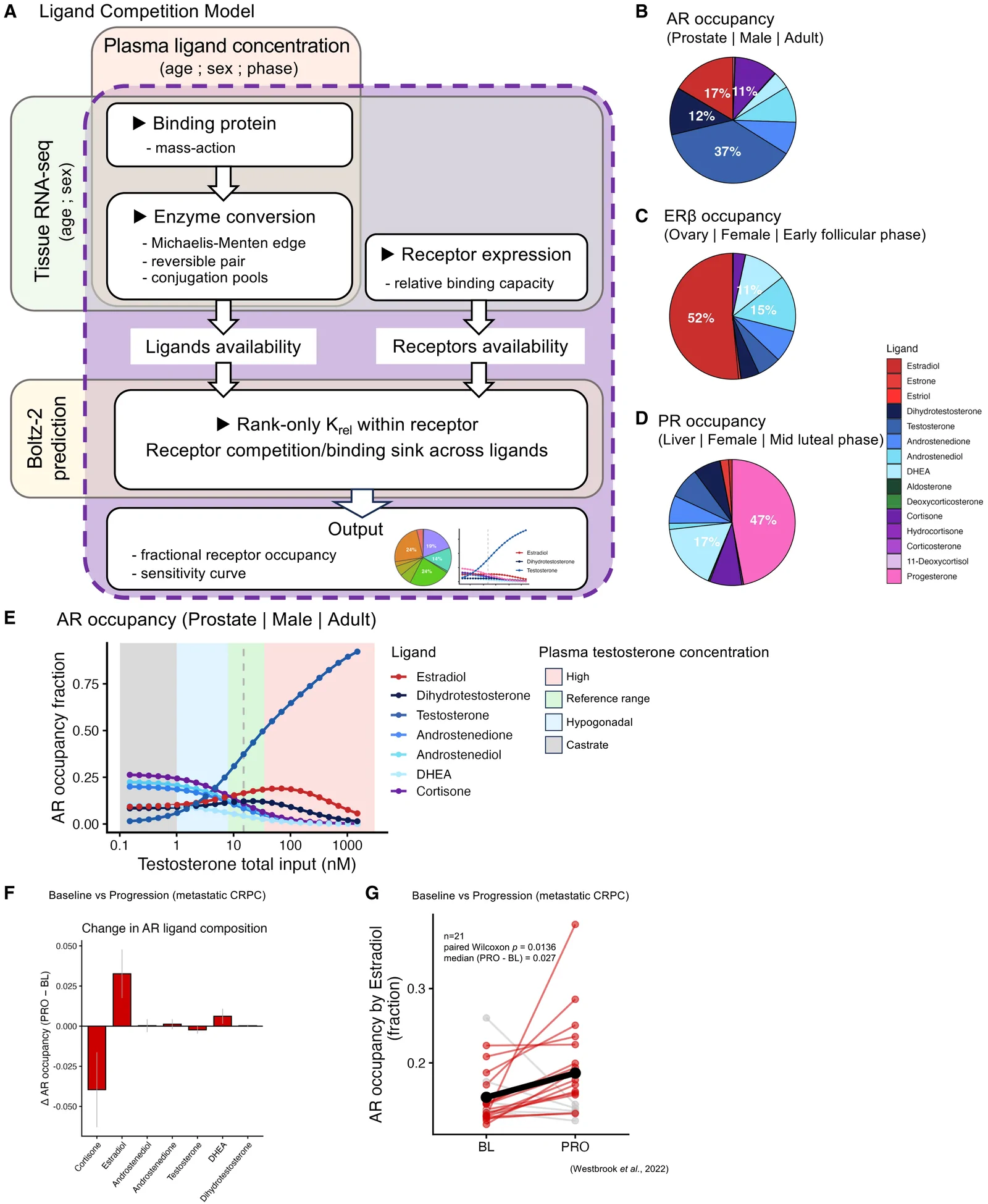
Physiologically constrained ligand competition contextualizes predicted receptor-ligand compatibility (A) Schematic overview of the ligand competition framework used to estimate receptor occupancy. Plasma steroid concentrations stratified by sex, age, and endocrine phase are combined with tissue-specific expression data from GTEx v.11 to model ligand availability and receptor abundance. Ligand pools are further modulated by binding-protein buffering (e.g., albumin, SHBG, and CBG) and enzymatic interconversion among steroid pathways (e.g., aromatase, 5α-reductase, and 11β-hydroxysteroid dehydrogenases). Boltz-2 predictions are incorporated as rank-based relative affinity scores within each receptor, and ligands compete for receptor occupancy in proportion to concentration divided by relative affinity. (B–D) Predicted fractional ligand contributions to receptor occupancy under representative physiological conditions. (B) AR occupancy in adult male prostate tissue. (C) ERβ occupancy in ovary during the early follicular phase. (D) PR occupancy in female liver during the mid-luteal phase. (E) AR occupancy across a testosterone sweep in adult male prostate. Total plasma testosterone input was varied over a log-scaled range while all other plasma ligand concentrations and tissue parameters were held constant. For each condition, ligand-specific AR occupancy fractions were recalculated using the same rank-based Boltz-2-derived compatibility matrix together with plasma binding, tissue redistribution, and enzyme conversion steps in the prostate model. Shaded regions indicate clinically relevant testosterone ranges: castrate (0.1–1 nM), hypogonadal (1–8 nM), adult male reference range (20–49 years; 8–35 nM), and high (>35 nM), and the dashed line marks the baseline (BL) testosterone input used in the reference male condition. (F) Predicted 17β-estradiol (estradiol) contribution to AR occupancy in metastatic castration-resistant prostate cancer (CRPC). Tumor-specific enzyme-expression profiles from paired BL and progression (PRO) biopsies on enzalutamide therapy were substituted for GTEx tissue drivers in the same rank-based endocrine competition model. Bars indicate mean ± SEM change in fractional AR occupancy for each ligand (PRO − BL). Estradiol shows the largest positive shift across ligands, suggesting that tumor-associated endocrine reprogramming preferentially increases its relative contribution to AR occupancy under a common androgen-deprived plasma background. (G) Predicted estradiol contribution to AR occupancy in matched CRPC samples before treatment (BL) and at progression on enzalutamide therapy (PRO). Lines connect paired patient samples; the black line indicates the mean trend. The model predicts a significant increase in estradiol-derived AR occupancy after progression, consistent with enhanced estradiol contribution to AR engagement in therapy-resistant tumors.

To explore how the predicted compatibility landscape may be interpreted under these competitive conditions, we examined ligand occupancy patterns within a simplified competition model incorporating physiological plasma steroid concentrations, circulating binding proteins, and tissue-specific receptor and steroid-processing enzyme expression inferred from GTEx data (Figure 5A; Figures S2A and S2B). Ligand availability was adjusted through two contextual layers: circulating binding proteins (e.g., SHBG and corticosteroid-binding globulin) and tissue-local enzyme expression (including aromatase, 5α-reductases, and hydroxysteroid dehydrogenases). Receptor expression levels then defined tissue-specific ligand engagement capacity. Under typical adult male hormone concentrations, AR occupancy in prostate was dominated by androgens, including DHT and testosterone, with E2 contributing a minor fraction (Figure 5B). ERβ occupancy in ovary during the early follicular phase was dominated by estradiol (Figure 5C), whereas PR engagement in the liver during the mid-luteal phase was driven largely by progesterone (Figure 5D). Varying individual ligand-availability inputs did not alter the dominant ligand assignment across these conditions (Figure S2C).

We next examined how larger endocrine shifts altered ligand contributions. Increasing testosterone progressively shifted AR occupancy toward androgen ligands; however, E2 showed the largest relative increase in AR engagement under testosterone excess (Figure 5E). This effect arises from E2’s measurable compatibility with AR together with androgen-driven metabolism, in which elevated testosterone provides additional substrate for aromatase-mediated conversion to E2. Although E2 remains a minor contributor to total AR occupancy, its engagement scales with androgen abundance.

Finally, we explored a disease-associated scenario in which multiple determinants of the competition are remodeled simultaneously. We substituted GTEx-derived tissue parameters with the profiles from castration-resistant prostate cancer (CRPC) biopsies obtained before treatment and after progression on the AR inhibitor enzalutamide.^22^ Under a common androgen-deprived plasma background, the progression profiles produced shifts in predicted AR ligand composition within the model. E2 showed the largest increase in predicted AR occupancy after progression (Figure 5F), and this shift was consistently observed across matched patient samples (Figure 5G). These simulations illustrate how the same compatibility prediction can translate into different occupancy profiles when placed in distinct biological contexts.

Together, these analyses show that predicted E2 compatibility with AR must be interpreted in the context of ligand abundance, binding proteins, receptor expression, and tissue state. The competition model provides a simple framework for evaluating the potential ligand contributions within the specified assumptions. By combining a systematically generated compatibility landscape with a ligand competition model, the approach helps place computational predictions within a physiological context.

## Discussion

### Asymmetric E2 cross-compatibility as an organizing principle of NR3 signaling

E2 shows consistently high predicted compatibility across multiple NR3 receptors (AR, PR, GR, and MR), whereas canonical non-aromatized ligands show minimal reciprocal engagement with ERα and ERβ. This one-way cross-reactivity offers a coherent biochemical explanation for long-standing context-dependent observations in steroid endocrinology, particularly the paradox in which estradiol represses AR activity in healthy tissues yet can activate AR signaling in malignancy. This directionality suggests that NR3 receptors retain ancestral compatibility with E2, while later receptors evolved increasing specificity for upstream steroids.

### Estradiol engages a conserved AR pocket, and mutations amplify rather than invent compatibility

A key structural implication is that E2 does not require a specialized or mutation-exclusive binding mode to be accommodated by AR. Instead, docking places E2, testosterone, and DHT in overlapping regions of the canonical pocket (Figures 2A and 2B). Modeling of the clinically relevant AR T877A mutation indicates only modest potentiation of the pre-existing predicted affinity landscape across ligands, with E2 remaining among the higher ranked ligands (Figures 3A–3C). This pattern is consistent with disease-associated tuning of a latent compatibility landscape, but functional activation requires direct experimental testing in defined mutation and cofactor contexts.

### Evolutionary origin: Estradiol as an ancient ligand retained across NR3 diversification

The directionality of cross-reactivity is naturally explained by the evolutionary history of NR3 receptors. Phylogenetic and functional reconstructions support that the earliest steroid receptor was estrogen sensitive^5^^,^^23^ and that AR, PR, GR, and MR emerged later through duplication and divergence events in early vertebrates.^5^^,^^24^^,^^25^ Because these younger receptors inherited their ligand-binding scaffold from an estrogen-sensitive ancestor,^5^ residual estradiol compatibility may persist even as specificity for non-aromatized ligands evolves.

Our ancestral receptor analysis (Figure 4) is compatible with this evolutionary logic. AncSR1 is estrogen responsive in predicted affinity, consistent with experimentally supported ancestral estrogen sensitivity, while AncSR2 shows a broadened predicted affinity landscape, including multiple non-aromatized steroids. Across the affinity heatmap (Figure 4D), E2 maintains high predicted compatibility across ancestral and modern receptors, whereas other steroids show more diversified receptor preferences following NR3 lineage expansion. Although estradiol is a terminal product of the steroidogenic cascade, it predates strict specialization of modern NR3 receptors, and its structural features remain compatible with the conserved NR3 pocket. In contrast, ERs remained specialized for aromatized estrogen recognition and did not show comparable predicted compatibility with non-aromatized steroids in the present screen, producing the asymmetric pattern observed across extant receptors.

### A two-state model: Estradiol as an end-product buffer in steroid physiology and cancer as a pathological exploit

**Figure 6:**
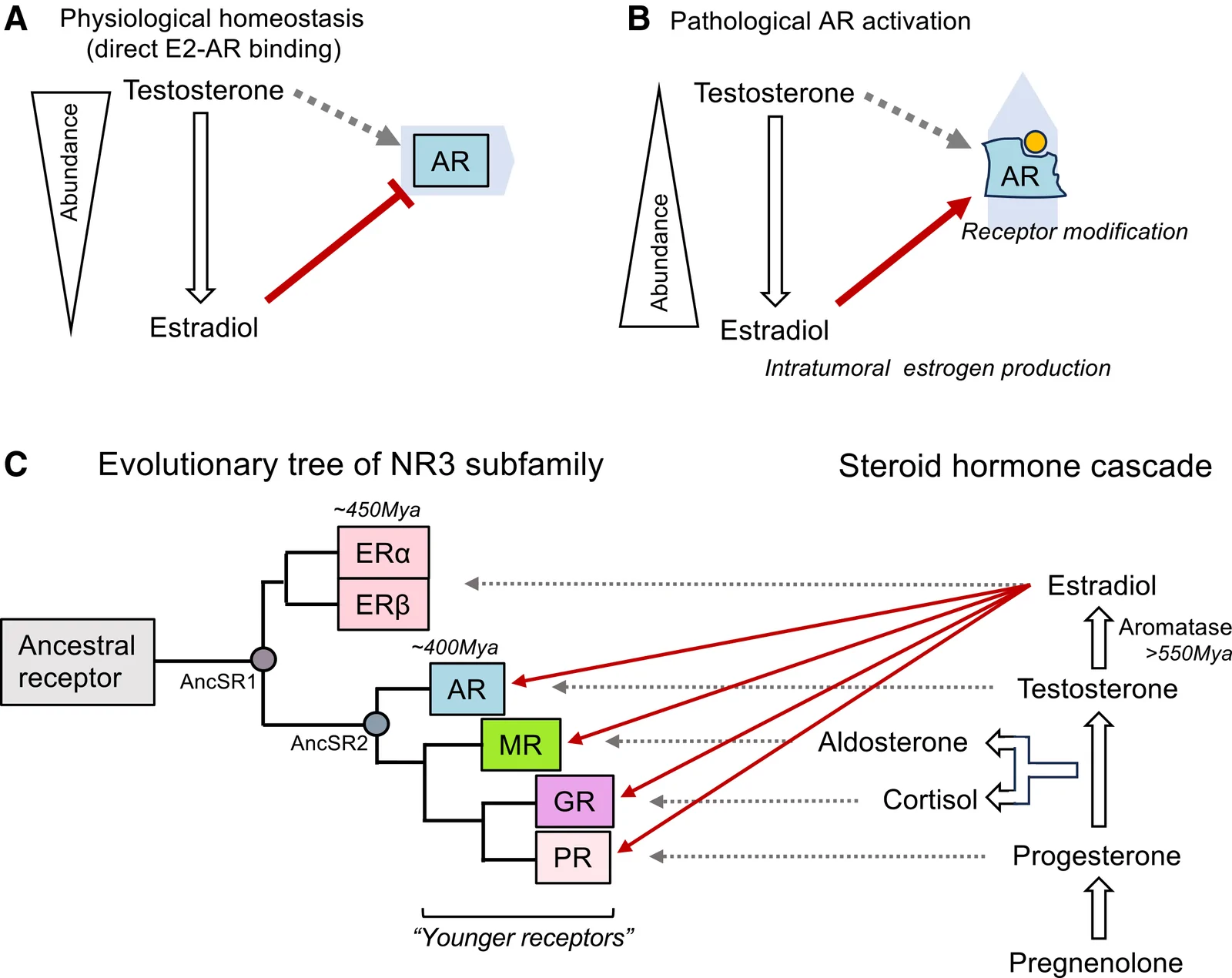
Conceptual model for evolutionary inheritance and pathological repurposing of 17β-estradiol-NR3 compatibility (A) Under normal endocrine conditions, circulating testosterone is present at substantially higher concentrations than estradiol, and AR signaling is primarily androgen driven. In the proposed buffering model, estradiol engages AR through an evolutionarily retained binding architecture and biases the receptor toward destabilization and reduced transcriptional output. This creates a unidirectional modulatory influence in which estradiol functions as a buffering brake on excessive androgen signaling. (B) In pathological contexts such as castration-resistant prostate cancer, receptor modification (e.g., AR ligand-binding domain mutations or altered co-regulator environments) can repurpose this retained estradiol compatibility. In the proposed disease-state model, estradiol engagement shifts from a buffering interaction toward productive receptor signaling, allowing estradiol to contribute to AR activity under conditions of limited androgen availability. (C) Phylogenetic reconstruction of the NR3 subfamily illustrates that the earliest steroid receptor (AncSR1) was estrogen sensitive. Subsequent duplication events produced ERα/β and later AncSR2, the common ancestor of AR, PR, GR, and MR. As receptor specialization proceeded, responsiveness to non-aromatized steroids diversified while compatibility with estradiol was retained across multiple NR3 lineages. The steroidogenic pathway proceeds from pregnenolone through progesterone and androgens to estradiol via aromatization (CYP19A1), which predates diversification of modern NR3 receptors. This evolutionary framework provides a potential explanation for the asymmetric compatibility pattern identified in the present study, in which estradiol remains compatible with multiple NR3 receptors whereas upstream steroids do not show comparable compatibility with estrogen receptors.

The persistence of predicted E2 compatibility across NR3 receptors suggests a hypothesis for future study. We propose a two-state model in which the consequences of this compatibility depend on biological context. Under physiological conditions, E2 may function as a downstream modulatory signal within a shared steroid receptor architecture (Figure 6A). In contrast, pathological states may exploit this latent compatibility to reshape receptor engagement and signaling outcomes (Figure 6B).

Steroid hormones share a common cholesterol-derived biosynthetic cascade, raising the possibility that an aromatized end product could provide modulatory input to receptors that evolved from an estrogen-sensitive ancestral architecture. E2 may engage AR without generating robust transcriptional output, consistent with prior evidence that E2-bound AR can be inefficient at coactivator recruitment and downstream activation.^7^ Reports that estradiol promotes AR destabilization and proteasomal degradation^6^ and that developmental estrogen exposure can reduce AR protein in prostate tissue^6^^,^^26^ provide biological precedent for this possibility. We therefore propose a buffering model in which estradiol may engage receptors that evolved specificity for upstream steroid intermediates under specific physiological contexts (Figure 6A). In this framework, asymmetric compatibility would permit downstream modulation of androgenic, progestational, or corticoid signaling while minimizing reciprocal interference from abundant upstream steroids with estrogen receptor function.

Malignancies may exploit this architecture (Figure 6B). Prior work showed that the AR coactivator ARA70 can enable estradiol-driven AR transcriptional activation^7^ and that altered coactivator landscapes in advanced disease can lower the barrier for noncanonical AR activation.^27^ Clinically, ligand-binding domain mutations further expand AR permissiveness in a subset of castration-resistant prostate cancers,^28^^,^^29^^,^^30^ including variants in which estradiol can stimulate AR target gene expression, consistent with disease exploiting latent receptor properties embedded by evolutionary history. Importantly, receptor engagement *in vivo* is not determined by binding affinity alone but also by ligand availability, receptor abundance, co-regulator state, and tissue context.^31^^,^^32^^,^^33^ Aromatase expression in prostate tumors increases the intratumoral estrogen availability.^34^ We therefore present pathological repurposing of latent estradiol compatibility as a disease-context hypothesis that may help explain heterogeneous responses to endocrine therapies.^35^^,^^36^

Together, these factors provide a plausible route by which basal E2 compatibility with AR could be co-opted in permissive disease states, while this remains a hypothesis for targeted biochemical and cellular validation.

### Extending the buffering concept across NR3: PR, GR, and MR as parallel substrates for estradiol engagement

Although AR provides a clinically salient example, the same conserved architecture may extend across NR3 receptors. ERs and AR represent the two major branches of the NR3 family, indicating that the observed asymmetry spans the principal NR3 lineages. Our computational survey suggests predicted E2 compatibility with PR, GR, and MR (Figures 1G–1I), consistent with descent from an estrogen-sensitive ancestor.^5^ This supports a broader principle: estradiol, as an aromatized end product, may act as a system-level regulator of upstream steroid axes through direct receptor engagement (Figure 6C). Asymmetric compatibility would permit estradiol to modulate androgenic, progestational, or corticoid signaling while minimizing reciprocal interference from abundant upstream steroids with estrogen receptor function.

In PR biology, estrogen-progesterone antagonism is typically interpreted through receptor crosstalk and transcriptional network effects.^37^^,^^38^ The predicted E2-PR interaction suggests a possible additional hypothesis for context-dependent progesterone modulation. For GR and MR, no direct evidence currently demonstrates estradiol binding as a physiological ligand, although weak cross-compatibility remains structurally plausible. The MR accommodates multiple corticosteroids and progesterone within its ligand-binding pocket,^39^ and progesterone binds MR with high affinity despite not being a corticoid ligand.^40^ Evolutionary analyses further show that ancestral corticoid receptors evolved structural features that excluded estrogens.^41^ Thus, predicted E2 compatibility across NR3 receptors is best treated as a latent architectural feature inherited from a shared evolutionary origin. Whether this compatibility becomes functionally relevant is likely to depend on receptor state, endocrine context, and disease-associated conditions, as exemplified by the AR.

### Testable predictions and outlook

This framework generates several testable predictions. Tumors that activate AR through estradiol under permissive states, including those harboring promiscuous AR mutations such as T877A or H874Y,^8^^,^^28^^,^^30^ should be selectively vulnerable to interventions that disrupt the E2-AR binding while preserving androgen-directed pharmacology. Because this activation requires a permissive cofactor environment, reducing ARA70/NCOA4 or related coactivators is predicted to convert estradiol from an agonist to a destabilizing or antagonistic ligand.^6^^,^^7^ Tumors with elevated local estrogen production are also predicted to show reduced responses to AR-only strategies and may exhibit state-dependent sensitivity to aromatase inhibition,^42^ consistent with heterogeneous clinical outcomes.

We identify a computationally predicted asymmetric compatibility pattern within NR3 steroid receptors in which E2 retains compatibility with later-evolved receptors while reciprocal compatibility of upstream steroids with ERs is limited under the same screening conditions. Integrating compatibility prediction, structural modeling, mutation analysis, ancestral reconstruction, and physiological contextualization, we propose that broad E2 compatibility may reflect an evolutionarily retained architectural feature that can be investigated in physiological and disease contexts. This work demonstrates a large-scale strategy for mapping receptor-ligand landscapes, identifying family-level patterns, and prioritizing hypotheses for targeted experimental follow-up.

### Limitations of the study

Several limitations warrant consideration. Boltz-2 provides a scalable affinity landscape but does not replace biochemical binding measurements or functional assays. The model also cannot distinguish between binding modes that may differ in functional outcome. Predicted affinity alone does not determine transcriptional output, emphasizing the need for experimental validation in defined cofactor and mutation contexts. Finally, ancestral receptor reconstructions, although based on robust phylogenetic methods, carry uncertainty regarding the precise functional states of ancient proteins.

## Resource availability

### Lead contact

Requests for further information and resources should be directed to and will be fulfilled by the lead contact, Akhilesh B. Reddy (areddy@cantab.net).

### Materials availability

This study did not generate new unique reagents.

### Data and code availability

• Boltz-2 affinity prediction outputs generated in this study are provided in Data S1 and Data S2. Publicly available datasets analyzed in this study include the GTEx Consortium dataset and the European Genome-phenome Archive: EGAS00001005954.
• Custom code used for data processing, analysis, and figure generation is publicly available at https://github.com/ReddysLab/Yamazaki_iScience_NR_analysis.
• Any additional information required to reanalyze the data reported in this paper is available from the lead contact upon request.

## Acknowledgments

A.B.R. acknowledges funding from the 10.13039/100007928Perelman School of Medicine, University of Pennsylvania, and the 10.13039/100007929Institute for Translational Medicine and Therapeutics (ITMAT) at the University of Pennsylvania. This work was also supported by NIH DP1DK126167 and R35GM161590.

## Author contributions

S.Y. performed the data analysis and wrote the manuscript. A.B.R. designed the project and analyzed the results, secured funding, and wrote the paper with contributions from S.Y. Both authors have reviewed the final manuscript and agree on its interpretation.

## Declaration of interests

The authors declare no competing interests.

## Declaration of generative AI and AI-assisted technologies in the writing process

During the preparation of this work, the authors used ChatGPT (OpenAI) in order to improve readability. After using this tool, the authors reviewed and edited the manuscript as needed and take full responsibility for the content of the published article.

## STAR★Methods

### Key resources table

**Table:** 

REAGENT or RESOURCE	SOURCE	IDENTIFIER
**Deposited data**		

Nuclear receptor ligand affinity predictions (Data S1)	This paper	Data S1
Ligand-binding domain analysis and benchmarking	This paper	Data S2
GTEx v11 RNA-seq data	GTEx Consortium^43^	https://gtexportal.org
Castration-resistant prostate cancer RNA-seq dataset	Westbrook et al.^22^	EGAS00001005954; https://ega-archive.org/studies/EGAS00001005954
UniProt protein sequences	UniProt Consortium	https://www.uniprot.org
PubChem ligand structures	PubChem	https://pubchem.ncbi.nlm.nih.gov

**Software and algorithms**		

Boltz-2 (v2.2.1)	Passaro et al.^10^	https://github.com/jwohlwend/boltz
AutoDock Vina (v1.2.0)	Trott & Olson^44^; Eberhardt et al.^45^	https://github.com/ccsb-scripps/AutoDock-Vina
UCSF ChimeraX	Pettersen et al.^46^	https://www.rbvi.ucsf.edu/chimerax
R (v4.4.0)	R Foundation for Statistical Computing	https://www.r-project.org
ggplot2 (v4.0.0)	Wickham et al.^47^	https://ggplot2.tidyverse.org
Custom analysis code	This paper	https://github.com/ReddysLab/Yamazaki_iScience_NR_analysis

### Method details

#### Nuclear receptor sequence selection

Human nuclear receptors (48 receptors in total; Table S1) were included in an unbiased computational screen. Full-length canonical protein sequences were obtained from UniProt and used without truncation because full-length sequence input is the native Boltz-2 use case and allowed a uniform strategy across the receptor family. We recognize that ligand binding is mediated primarily by the ligand-binding domain (LBD) and that disordered or regulatory regions can introduce modeling uncertainty.^48^^,^^49^ For this reason, full-length modeling is interpreted as a consistent screen for relative landscape patterns rather than as a substitute for LBD-focused structural or experimental validation. To examine the impact of receptor modification on ligand affinity landscapes, the androgen receptor T877A mutation was modeled in silico. This mutation was selected based on its established role in altering steroid responsiveness in prostate cancer.^50^ The mutant AR sequence was generated by single-residue substitution within the full-length receptor and subjected to the same all-by-all ligand screening pipeline as wild-type AR. Reconstructed ancestral nuclear receptor sequences (AncSR1 and AncSR2) were obtained from previously published phylogenetic analyses of the NR3 lineage.^4^^,^^20^ These sequences were derived using maximum-likelihood methods and experimentally evaluated in prior work. Ancestral receptors were treated equivalently to extant receptors in all computational analyses.

#### Ligand library construction

This comprehensive collection includes all major endogenous nuclear hormone receptor ligands, including steroid hormones, thyroid hormones, vitamin D metabolites, retinoids, fatty acids, and bile acids (Table S2). All ligand identities were verified through systematic PubChem searches, and all PubChem compound identifiers (CIDs) were cross-checked. Ligands were not pre-assigned to cognate receptors.

#### Ligand-receptor affinity prediction using Boltz-2

Ligand-receptor interactions were predicted using Boltz-2 (v2.2.1), a deep learning modeling framework trained on experimentally determined biomolecular structural data.^10^ Data were generated on a compute node with 8 Tesla P40 Nvidia GPUs (24 GB each), 512 GB RAM, and 4 TB SSD. For each receptor-ligand pair, we used the primary Boltz-2 outputs affinity_pred_value and affinity_probability_binary. According to the Boltz-2 framework, affinity_pred_value represents the model-predicted log_10_(IC_50_ [μM]), which was converted to IC_50_ (nM) for presentation. affinity_probability_binary represents the model-estimated probability that a ligand is a binder rather than a decoy and was used to define high-confidence predictions. Unless otherwise specified, receptor-ligand pairs with affinity_probability_binary ≥0.90 were considered high-confidence for visualization. All receptors were screened against the complete ligand library, generating an unbiased all-by-all interaction matrix. Predicted IC_50_ values were used for within-receptor and related-receptor ranking, not as experimentally measured binding constants. Structural confidence metrics (including confidence_score, pTM, ipTM, ligand_ipTM, protein_ipTM, and complex_pLDDT) were retained as model-quality descriptors but were not used for affinity estimation, ligand ranking, or binder classification. The complete prediction matrix, including displayed and non-displayed receptor-ligand pairs, is provided as Data S1.

Representative published competitive-binding IC_50_ measurements were obtained for ERα, ERβ, AR, PR, GR, and MR and compared with the corresponding Boltz-2 predictions (Table S3). To minimize inter-assay variability, each receptor was represented by measurements from a single publication and internally consistent experimental condition. Canonical ligands and available noncanonical ligands present in the manuscript ligand panel were included where reported. Because Boltz-2 IC_50_ estimates were interpreted as relative affinity scores rather than calibrated absolute concentrations, experimental and predicted log_10_(IC_50_) values were centered separately within each receptor by subtracting the receptor-specific mean. Spearman rank correlation was calculated separately for each receptor without cross-receptor pooling.

LBD-only Boltz-2 predictions were generated for the six human steroid receptors (ERα, ERβ, AR, PR, GR, and MR) using UniProtKB LBD annotations (Data S2). The same 54-ligand panel and Boltz-2 workflow used for the full-length analyses were applied to the isolated LBD sequences. Predicted IC_50_ and pIC_50_ values were derived from affinity_pred_value as described above and matched to the corresponding full-length predictions by receptor and ligand. Agreement between full-length and LBD predictions was evaluated separately for each receptor using Spearman’s ρ and Kendall’s τ across the complete 54-ligand panel.

#### Molecular docking and structural comparison

To independently evaluate whether screen-prioritized ligands could be accommodated within the known AR ligand-binding architecture, 17β-estradiol, testosterone, and dihydrotestosterone were docked into the androgen receptor using AutoDock Vina.^44^^,^^45^ Docking was performed using a cubic search grid (40 × 40 × 40 Å) centered on the receptor center of mass and encompassing the canonical AR ligand-binding cavity defined in prior structural studies.^51^ For each ligand, the highest-scoring pose located within the canonical binding pocket was selected. To validate the docking protocol, the crystallographic AR-DHT complex (PDB: 1I37) was used for ligand redocking. After removing the crystallographic DHT ligand, it was redocked using AutoDock Vina. The top-ranked pose accurately reproduced the experimental binding orientation (symmetry-corrected heavy-atom RMSD = 0.38 Å), validating the docking procedure (Figure S1C). Heavy-atom RMSD was calculated using symmetry-equivalent atom mappings without ligand-only coordinate superposition. Docking scores were similar across ligands (17β-estradiol: −7.37 kcal/mol; testosterone: −7.52 kcal/mol; dihydrotestosterone: −7.48 kcal/mol). Pose centroid distances were 0.624 Å for 17β-estradiol versus testosterone and 0.829 Å for 17β-estradiol versus dihydrotestosterone. Structural visualization and superposition were performed using ChimeraX.^46^ These docking analyses were used only to assess structural compatibility and shared-pocket accommodation, not to infer physiological binding, activation, or functional equivalence.

#### Evolutionary integration and comparative analysis

Predicted ligand affinities for extant and ancestral receptors were interpreted in the context of established NR3 phylogeny and steroid biosynthetic pathways. Divergence timing and ancestral functional states were based on prior evolutionary analyses.^20^ Steroid biosynthetic relationships were overlaid conceptually to relate predicted receptor affinities to the emergence of enzymatic steps such as aromatization by CYP19A1.^21^

#### Ligand competition analysis

To estimate receptor occupancy in physiological endocrine contexts, predicted ligand affinities were integrated with circulating hormone concentrations and tissue receptor expression. Competitive receptor occupancy was approximated using a concentration-affinity relationship derived from classical ligand-binding models.^52^^,^^53^ Physiological hormone concentrations were based on established endocrine reference ranges.^54^^,^^55^ Inputs include circulating ligand concentrations, rank-derived relative affinity terms, binding-protein adjustment, tissue receptor transcript abundance, and selected steroidogenic enzyme expression. Key assumptions are that Boltz-2 rankings can be converted into within-receptor relative compatibility terms, circulating concentrations approximate ligand availability, and transcript abundance provides contextual rather than direct protein or flux measurements. Outputs are fractional ligand contributions within the specified simplified context.

For each receptor *R* and ligand *L*, a binding drive term was calculated as:

(Equation 1) $$W_{R,L}=\frac{C_{L}}{{IC50}_{R,L}}$$

where *C*_*L*_ is the circulating ligand concentration and *IC*50_*R*,*L*_ is the Boltz-2-predicted affinity.

Fractional competitive occupancy was then calculated as:

(Equation 2) $${Occ}_{R,L}=\frac{W_{R,L}}{\sum_{L}\;W_{R,L}}$$

which represents the relative ligand composition predicted to occupy each receptor under the specified endocrine conditions.

To approximate tissue-level receptor context, receptor expression values from GTEx v11 bulk RNA-seq datasets (ages 20–49 for both males and females)^43^ were used as tissue-state descriptors rather than direct protein-abundance measurements. For tissue t, predicted ligand occupancy was weighted by receptor expression to estimate a contextual binding sink:

(Equation 3) $${Bound}_{t,R,L}={TPM}_{t,R}\times {Occ}_{R,L}$$

where *TPM*_*t*,*R*_ represents the median transcript abundance of receptor *R* in tissue *t*. These values were aggregated across receptors to estimate tissue-level nuclear receptor ligand engagement.

In extended analyses, tissue-specific steroid metabolism was approximated using expression of key steroidogenic and metabolic enzymes (CYP11A1, CYP17A1, CYP19A1, SRD5A1/2, HSD11B1/2, STS, SULT1E1, UGT2B7/15/17) together with circulating hormone-binding proteins (SHBG, SERPINA6, albumin;^56^^,^^57^). These factors were used to proportionally redistribute ligand availability prior to occupancy calculations. Because mRNA abundance does not directly measure enzyme activity, metabolic flux, protein concentration, or co-regulator state, these terms are treated as contextual modifiers rather than validated physiological parameters.

For disease-context modeling, receptor and enzyme expression values were replaced with RNA-seq measurements from castration-resistant prostate cancer samples,^22^ enabling comparison between physiological and tumor endocrine environments under a common exploratory framework. Model outputs were exported as receptor × ligand × tissue occupancy matrices for downstream visualization and comparative analysis.

#### Data visualization and analysis

All figures and supporting analyses were generated in R (version 4.4.0) using ggplot2 (version 4.0.0).^47^ Bar plots display predicted log_10_(IC_50_) values ordered by affinity, with color intensity representing prediction confidence. Scatterplots comparing wild-type and mutant AR affinities were plotted on matched log scales, with the diagonal indicating equal predicted affinity.

### Quantification and statistical analysis

This study primarily involved computational prediction, ranking, and comparative analysis of receptor–ligand interaction landscapes. Statistical procedures used for individual analyses are described in the corresponding figure legends. Predicted affinity values are reported as Boltz-2-generated IC_50_ estimates and were used for comparative ranking and visualization rather than as experimentally measured binding constants.

Where applicable, quantitative comparisons were performed using R (version 4.4.0). Statistical tests, significance thresholds, and sample definitions are reported in the corresponding figures and legends.

## Supplemental information

Document S1. Figures S1 and S2 and Tables S1–S3

Data S1. Predicted ligand-receptor affinity matrix for all human nuclear receptors and ligands analyzed, including IC_50_ estimates and confidence scores

Data S2. Ligand-binding domain definitions, full-length and isolated ligand-binding domain Boltz-2 prediction results, and receptor-specific correlation statistics for ERα, ERβ, AR, PR, GR, and MR
