# Supplementary material for: Asymmetric cross-reactivity of nuclear receptors reveals an evolutionary buffer between estrogen and androgen signaling
Source: iScience. 2026 Aug 19;29(9):117289. doi: 10.1016/j.isci.2026.117289 (PMC13521070; doi:10.1016/j.isci.2026.117289)
Supplement: Supplementary file 1 [file mmc1.pdf]

iScience, Volume *29*

Supplemental information

Asymmetric cross-reactivity of nuclear receptors reveals an evolutionary buffer between estrogen and androgen signaling

Shintaro Yamazaki and Akhilesh B. Reddy

Figure S1. Validation of Boltz-2 predictions and molecular docking.

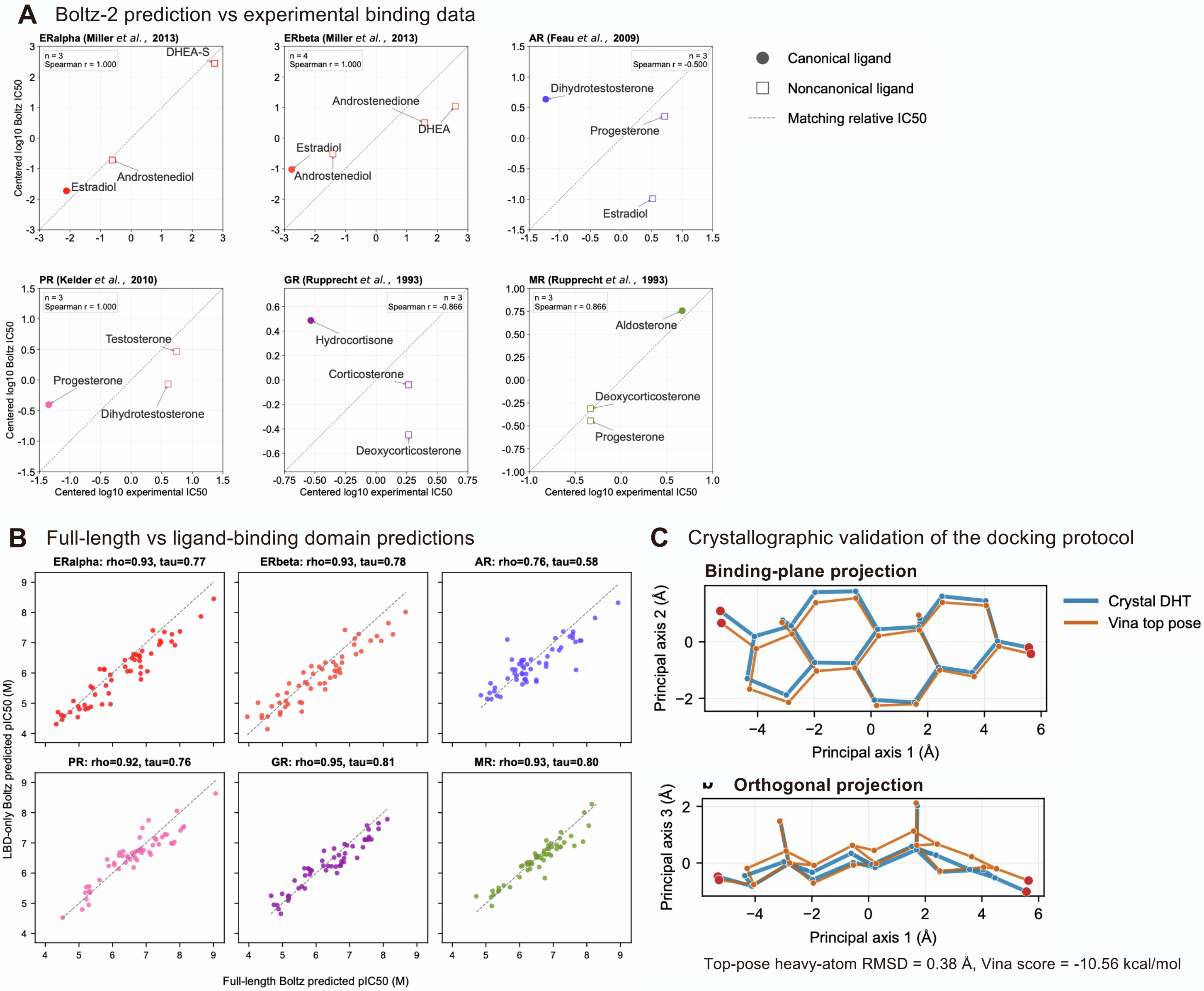

Figure S1. Validation of Boltz-2 predictions and molecular docking.
(A) Boltz-2 predictions versus experimental binding data. Representative published competitive-binding IC_{50} values from a single internally consistent study per receptor were compared with corresponding Boltz-2 predictions. Experimental and predicted $\log_{10}(IC_{50})$ values were centered separately on the receptor-specific geometric mean to compare relative ligand potency without implying absolute IC_{50} calibration. Filled circles denote canonical ligands and open squares denote noncanonical ligands. The dashed diagonal indicates equal relative IC_{50} displacement from the receptor-specific geometric mean. Spearman's ρ and the number of ligands are shown for each receptor. (B) Full-length versus ligand-binding domain predictions. Full-length and LBD-only Boltz-2 predicted pIC_{50} values are shown for the same 54-ligand panel for ERα, ERβ, AR, PR, GR, and MR. Each point represents one receptor-ligand pair. The dashed diagonal indicates equality between full-length and LBD predictions. Spearman's ρ is shown for each receptor. (C) Crystallographic validation of the docking protocol. The top-ranked AutoDock Vina pose of DHT redocked into the human AR ligand-binding domain (PDB: 1I37) is compared with the crystallographic DHT orientation in binding-plane and orthogonal views. Blue denotes the crystallographic ligand and orange the redocked pose. The docking protocol reproduced the crystallographic binding mode with a symmetry-corrected heavy-atom RMSD of 0.38 Å and a Vina score of -10.56 kcal/mol.

Figure S2. Tissue receptor expression, steroidogenic enzyme expression, and ligand competition sensitivity analyses.

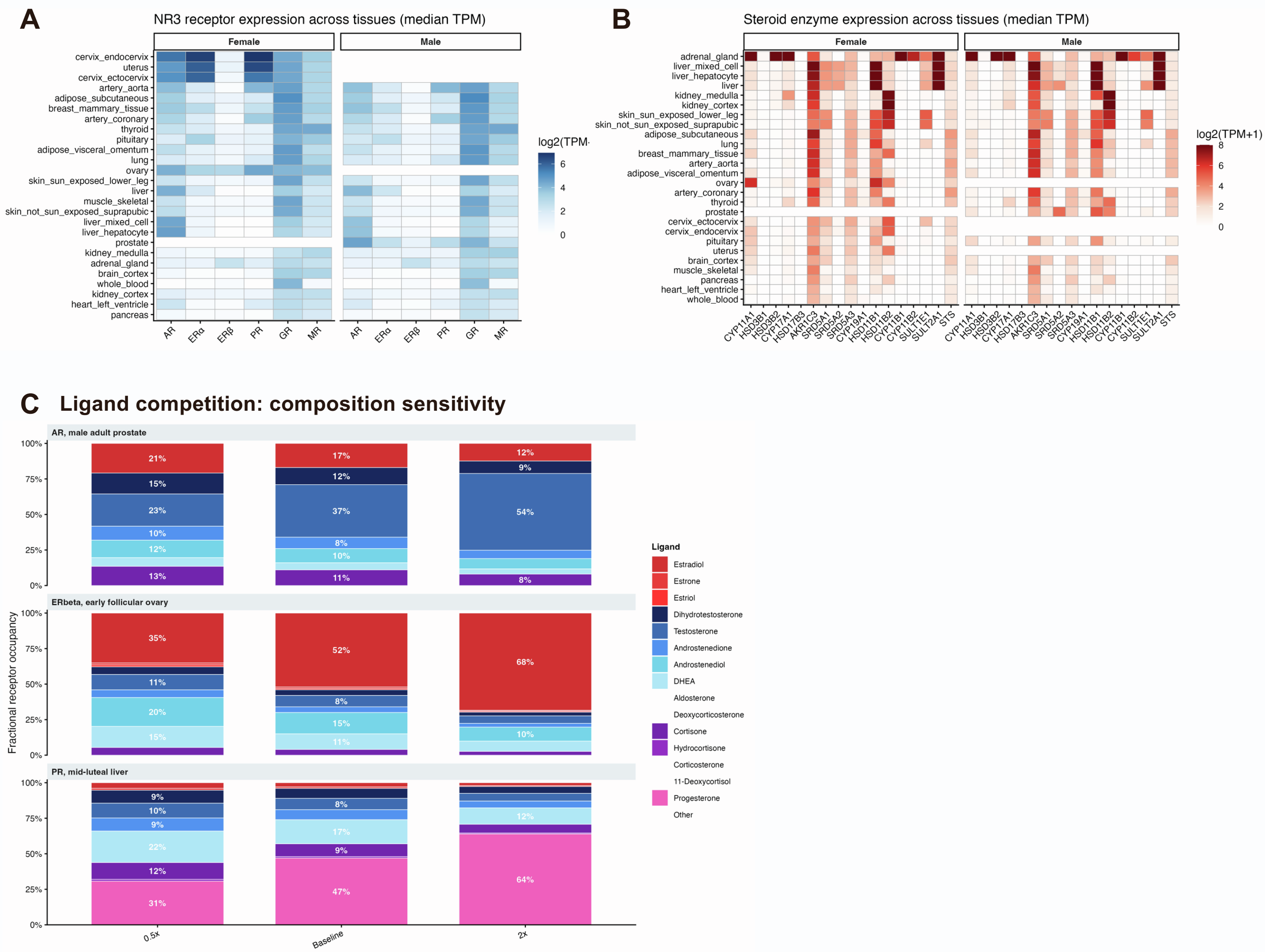

Figure S2. Tissue receptor expression, steroidogenic enzyme expression, and ligand competition sensitivity analyses.
(A) Expression levels of NR3 steroid receptors across human tissues based on GTEx v11 transcriptomic data. (B) Expression levels of key steroidogenic and steroid-metabolizing enzymes across the same tissue panel. Expression values represent normalized transcript abundance from GTEx v11. (C) Ligand competition model sensitivity analysis. Stacked bars show model-predicted fractional receptor occupancy at baseline and after varying the conventional ligand availability to 0.5× or 2× baseline. Baseline values correspond to the occupancy compositions shown in Figure 6B-D. Across this range, the dominant ligand identity remained unchanged, with testosterone, estradiol, and progesterone remaining the largest predicted contributors to AR, ERβ, and PR occupancy, respectively.

Table S1

List of the 48 canonical human nuclear hormone receptors included in the all-by-all affinity screen, with NR classifications, gene symbols and known functions.

NR Subfamily	NR Classification	Gene Symbol	Receptor Name	Function
NR0: Atypical receptors (lacking typical domains)	NR0B1	DAX1 (NR0B1)	DAX-1	Orphan receptor
NR0: Atypical receptors (lacking typical domains)	NR0B2	SHP (NR0B2)	Small heterodimer partner	Orphan receptor
NR1: Thyroid hormone-like receptors	NR1A1	THRA	Thyroid hormone receptor α	Thyroid hormone signaling
NR1: Thyroid hormone-like receptors	NR1A2	THRB	Thyroid hormone receptor β	Thyroid hormone signaling
NR1: Thyroid hormone-like receptors	NR1B1	RARA	Retinoic acid receptor α	Retinoic acid signaling
NR1: Thyroid hormone-like receptors	NR1B2	RARB	Retinoic acid receptor β	Retinoic acid signaling
NR1: Thyroid hormone-like receptors	NR1B3	RARG	Retinoic acid receptor γ	Retinoic acid signaling
NR1: Thyroid hormone-like receptors	NR1C1	PPARA	Peroxisome proliferator-activated receptor α	Lipid metabolism
NR1: Thyroid hormone-like receptors	NR1C2	PPARG	Peroxisome proliferator-activated receptor γ	Adipogenesis
NR1: Thyroid hormone-like receptors	NR1C3	PPARD	Peroxisome proliferator-activated receptor δ	Fatty acid oxidation
NR1: Thyroid hormone-like receptors	NR1D1	NR1D1	Rev-erb α	Circadian rhythm
NR1: Thyroid hormone-like receptors	NR1D2	NR1D2	Rev-erb β	Circadian rhythm
NR1: Thyroid hormone-like receptors	NR1F1	RORA	RAR-related orphan receptor α	Circadian rhythm
NR1: Thyroid hormone-like receptors	NR1F2	RORB	RAR-related orphan receptor β	Neural development
NR1: Thyroid hormone-like receptors	NR1F3	RORC	RAR-related orphan receptor γ	Immune function
NR1: Thyroid hormone-like receptors	NR1H2	NR1H2	Liver X receptor β	Cholesterol homeostasis
NR1: Thyroid hormone-like receptors	NR1H3	NR1H3	Liver X receptor α	Cholesterol homeostasis
NR1: Thyroid hormone-like receptors	NR1H4	NR1H4	Farnesoid X receptor	Bile acid metabolism
NR1: Thyroid hormone-like receptors	NR1I1	VDR	Vitamin D receptor	Calcium homeostasis
NR1: Thyroid hormone-like receptors	NR1I2	NR1I2	Pregnane X receptor	Xenobiotic metabolism
NR1: Thyroid hormone-like receptors	NR1I3	NR1I3	Constitutive androstane receptor	Xenobiotic metabolism
NR2: Retinoid X receptor-like receptors	NR2A1	HNF4A	Hepatocyte nuclear factor 4α	Hepatic function
NR2: Retinoid X receptor-like receptors	NR2A2	HNF4G	Hepatocyte nuclear factor 4γ	Hepatic function
NR2: Retinoid X receptor-like receptors	NR2B1	RXRA	Retinoid X receptor α	Heterodimerization partner
NR2: Retinoid X receptor-like receptors	NR2B2	RXRB	Retinoid X receptor β	Heterodimerization partner
NR2: Retinoid X receptor-like receptors	NR2B3	RXRG	Retinoid X receptor γ	Heterodimerization partner
NR2: Retinoid X receptor-like receptors	NR2C1	NR2C1	Testicular receptor 2	Testicular function
NR2: Retinoid X receptor-like receptors	NR2C2	NR2C2	Testicular receptor 4	Testicular function
NR2: Retinoid X receptor-like receptors	NR2E1	NR2E1	Tailless homolog	Neural development
NR2: Retinoid X receptor-like receptors	NR2E3	NR2E3	Photoreceptor cell-specific nuclear receptor	Retinal development
NR2: Retinoid X receptor-like receptors	NR2F1	NR2F1	COUP transcription factor 1	Development
NR2: Retinoid X receptor-like receptors	NR2F2	NR2F2	COUP transcription factor 2	Development
NR2: Retinoid X receptor-like receptors	NR2F6	NR2F6	COUP transcription factor 3	Development
NR3: Steroid hormone receptors	NR3A1	ESR1	Estrogen receptor α	Estrogen signaling
NR3: Steroid hormone receptors	NR3A2	ESR2	Estrogen receptor β	Estrogen signaling
NR3: Steroid hormone receptors	NR3B1	ESRRA	Estrogen-related receptor α	Metabolic regulation
NR3: Steroid hormone receptors	NR3B2	ESRRB	Estrogen-related receptor β	Metabolic regulation
NR3: Steroid hormone receptors	NR3B3	ESRRG	Estrogen-related receptor γ	Metabolic regulation
NR3: Steroid hormone receptors	NR3C1	NR3C1	Glucocorticoid receptor	Stress response
NR3: Steroid hormone receptors	NR3C2	NR3C2	Mineralocorticoid receptor	Electrolyte balance
NR3: Steroid hormone receptors	NR3C3	PGR	Progesterone receptor	Reproductive function
NR3: Steroid hormone receptors	NR3C4	AR	Androgen receptor	Male development
NR4: Nerve growth factor IB-like receptors	NR4A1	NR4A1	Nerve growth factor IB	Immediate early gene
NR4: Nerve growth factor IB-like receptors	NR4A2	NR4A2	Nuclear receptor related 1	Dopaminergic neurons
NR4: Nerve growth factor IB-like receptors	NR4A3	NR4A3	Neuron-derived orphan receptor 1	Neural function
NR5: Fushi tarazu-F1 like receptors	NR5A1	NR5A1	Steroidogenic factor 1	Steroidogenesis
NR5: Fushi tarazu-F1 like receptors	NR5A2	NR5A2	Liver receptor homolog 1	Hepatic function
NR6: Germ cell nuclear factor-like receptors	NR6A1	NR6A1	Germ cell nuclear factor	Germ cell development

Table S2
List of endogenous ligands used in the study.

category	ligand_name	pubchem_cid
STEROID PRECURSORS	Cholesterol	5997
STEROID PRECURSORS	Pregnenolone	8955
STEROID PRECURSORS	17α-Hydroxypregnenolone	91451
PROGESTOGENS	Progesterone	5994
PROGESTOGENS	17α-Hydroxyprogesterone	6238
PROGESTOGENS	5α-Pregnane-3,20-dione	92810
PROGESTOGENS	5β-Pregnane-3,20-dione	92745
PROGESTOGENS	Pregnane-3,20-diol	4901
ANDROGENS	Testosterone	6013
ANDROGENS	Dihydrotestosterone	10635
ANDROGENS	Androstenedione	6128
ANDROGENS	DHEA	5881
ANDROGENS	DHEA-S	12594
ANDROGENS	Androsterone	5879
ANDROGENS	Androsterone sulfate	159663
ANDROGENS	Epiandrosterone	441302
ANDROGENS	Epiandrosterone sulfate	9929317
ANDROGENS	Etiocholanolone	5880
ANDROGENS	Androstenediol	10634
ANDROGENS	3α-Androstanediol	15818
ANDROGENS	3β-Androstanediol	242332
ANDROGENS	Androstenol	101989
ESTROGENS	Estradiol	5757
ESTROGENS	Estrone	5870
ESTROGENS	Estriol	5756
GLUCOCORTICOIDS	Hydrocortisone	5754
GLUCOCORTICOIDS	Cortisone	222786
GLUCOCORTICOIDS	Corticosterone	5753
GLUCOCORTICOIDS	11-Deoxycortisol	440707
GLUCOCORTICOIDS	Tetrahydrocortisol	5864
GLUCOCORTICOIDS	Allotetrahydrocortisol	92748
GLUCOCORTICOIDS	Tetrahydrocortisone	5866
GLUCOCORTICOIDS	Tetrahydrodeoxycortisol	65555
MINERALOCORTICOIDS	Aldosterone	5839
MINERALOCORTICOIDS	18-Hydroxycorticosterone	11222
MINERALOCORTICOIDS	Deoxycorticosterone	6166
MINERALOCORTICOIDS	Tetrahydrodeoxycorticosterone	9974162
NEUROSTEROIDS	Allopregnanolone	92786
NEUROSTEROIDS	Pregnanolone	31402
THYROID HORMONES	T4	5819
THYROID HORMONES	T3	5920
THYROID HORMONES	Reverse T3	44123465
VITAMIN D METABOLITES	Vitamin D3	5280795
VITAMIN D METABOLITES	Vitamin D2	5280793
VITAMIN D METABOLITES	Calcidiol	5283731
VITAMIN D METABOLITES	Calcitriol	5280453
RETINOIDS	All-trans-Retinoic Acid	444795
RETINOIDS	9-cis-Retinoic Acid	449171
RETINOIDS	Retinol	445354
FATTY ACIDS	Palmitic Acid	985
FATTY ACIDS	Oleic Acid	445639
FATTY ACIDS	Linoleic Acid	5280450
FATTY ACIDS	Arachidonic Acid	444899
BILE ACIDS	Cholic Acid	221493
BILE ACIDS	Chenodeoxycholic Acid	10133
BILE ACIDS	Deoxycholic Acid	222528
BILE ACIDS	Lithocholic Acid	9903

Table S3

Comparison of Boltz-2 predictions with representative experimental receptor-ligand binding data.

Receptor	Ligand	Ligand class	Experimental value as reported	Experimental IC50 (nM)	Boltz predicted IC50 (nM)	Experimental rank	Boltz rank	Literature reference	Assay condition
ERalpha	Estradiol	canonical	approximately 1-10 nM	3.162277660168379	0.9779971973828678	1	1	Miller et al., Steroids 2013;78:15-25	competition against 30 nM [3H]estradiol; 1 h at 37 C; hydroxyapatite separation
ERalpha	Androstenediol	noncanonical	approximately 0.1 uM	100.0	9.914050031720198	2	2	Miller et al., Steroids 2013;78:15-25	competition against 30 nM [3H]estradiol; 1 h at 37 C; hydroxyapatite separation
ERalpha	DHEA-S	noncanonical	100-500 uM	223606.79774997896	14561.171779130156	3	4	Miller et al., Steroids 2013;78:15-25	competition against 30 nM [3H]estradiol; 1 h at 37 C; hydroxyapatite separation
ERalpha	DHEA	noncanonical	greater than 500 uM	500000.0	13.973786302132106	4	3	Miller et al., Steroids 2013;78:15-25	competition against 30 nM [3H]estradiol; 1 h at 37 C; hydroxyapatite separation
ERbeta	Estradiol	canonical	approximately 1-5 nM	2.23606797749979	2.176229326627681	1	1	Miller et al., Steroids 2013;78:15-25	competition against 30 nM [3H]estradiol; 1 h at 37 C; hydroxyapatite separation
ERbeta	Androstenediol	noncanonical	approximately 50 nM	50.0	7.057810699704288	2	2	Miller et al., Steroids 2013;78:15-25	competition against 30 nM [3H]estradiol; 1 h at 37 C; hydroxyapatite separation
ERbeta	Androstenedione	noncanonical	approximately 50 uM	50000.0	73.48690956156513	3	3	Miller et al., Steroids 2013;78:15-25	competition against 30 nM [3H]estradiol; 1 h at 37 C; hydroxyapatite separation
ERbeta	DHEA	noncanonical	500 uM	500000.0	256.851810595529	4	4	Miller et al., Steroids 2013;78:15-25	competition against 30 nM [3H]estradiol; 1 h at 37 C; hydroxyapatite separation
AR	Dihydrotestosterone	canonical	0.057 plus or minus 0.006 uM	57.0	49.96524290691037	1	3	Feau et al., Journal of Biomolecular Screening 2009;14:43-48	binding domain; 20 nM [3H]DHT; Ni-chelate FlashPlate SPA; optimized single protocol
AR	Estradiol	noncanonical	3.2 plus or minus 0.6 uM	3200.0	1.1756980846980496	2	1	Feau et al., Journal of Biomolecular Screening 2009;14:43-48	binding domain; 20 nM [3H]DHT; Ni-chelate FlashPlate SPA; optimized single protocol
AR	Progesterone	noncanonical	5.0 plus or minus 0.7 uM	5000.0	26.3638495928386	3	2	Feau et al., Journal of Biomolecular Screening 2009;14:43-48	binding domain; 20 nM [3H]DHT; Ni-chelate FlashPlate SPA; optimized single protocol
PR	Progesterone	canonical	7.74 nM	7.74	7.478948978149298	1	1	Kelder et al., Steroids 2010;75:314-322	human progesterone receptor under the report's single competitive-binding protocol
PR	Dihydrotestosterone	noncanonical	pIC50 6.16	691.8309709189363	16.243666375644477	2	2	Kelder et al., Steroids 2010;75:314-322	human progesterone receptor under the report's single competitive-binding protocol
PR	Testosterone	noncanonical	pIC50 6.02	954.992586021436	55.52927144968537	3	3	Kelder et al., Steroids 2010;75:314-322	human progesterone receptor under the report's single competitive-binding protocol
GR	Hydrocortisone	canonical	pIC50 8.0	10.0	119.4570040132314	1	3	European Journal of Pharmacology	human glucocorticoid receptor within one report
GR	Corticosterone	noncanonical	pIC50 7.2	63.09573444801943	35.52251700041934	2	2	European Journal of Pharmacology	human glucocorticoid receptor within one report
GR	Deoxycorticosterone	noncanonical	pIC50 7.2	63.09573444801943	13.8566708904591	2	1	European Journal of Pharmacology	human glucocorticoid receptor within one report
MR	Deoxycorticosterone	noncanonical	pIC50 11.0	0.01	11.88932735126454	1	2	European Journal of Pharmacology	human mineralocorticoid receptor within one report
MR	Progesterone	noncanonical	pIC50 11.0	0.01	8.750085768953024	1	1	European Journal of Pharmacology	human mineralocorticoid receptor within one report
MR	Aldosterone	canonical	pIC50 10.0	0.1	139.08424685377017	3	3	European Journal of Pharmacology	human mineralocorticoid receptor within one report
